# Global impact of parasitic infections and the importance of parasite control

**DOI:** 10.3389/fpara.2025.1546195

**Published:** 2025-03-10

**Authors:** Ronald Kaminsky, Pascal Mäser

**Affiliations:** ^1^ Para.C-Consulting, Altenstein, Germany; ^2^ Swiss Tropical and Public Health Institute, Allschwil, Switzerland; ^3^ University of Basel, Basel, Switzerland

**Keywords:** human parasites, animal parasites, parasite control, drug resistance, convenience of treatment, One Health

## Abstract

Parasites have a severe impact on animal and human health. Parasites like worms, ticks, mites, fleas, biting flies, mosquitoes, and pathogenic protozoa affect humans and their pets as well as their livestock globally, both in terms of severity and numbers. Parasitic infections are a global phenomenon, and they can be associated with severe or mild symptoms but represent a continuous risk of severe diseases for animals and humans. Therefore, effective treatment options and the prevention of infection are key for the wellbeing of pets, livestock, and humans, including the reduction of zoonotic risk of infection. The effective control of parasites in animals can greatly improve their quality of life and is also beneficial for humans; this is threatened by drug-resistant parasite populations. Today’s key areas for improvement of parasite control are as follows: a) convenience of prevention and treatment, b) effectiveness against drug-resistant parasites, c) availability and reduced costs of treatment, and d) control measurements that are environmentally friendly.

## 1 Introduction

Endoparasites like worms; ectoparasites like ticks, mites, fleas, biting flies, and mosquitoes; and pathogenic protozoa like leishmania, coccidia, and *Plasmodium* affect humans and their pets as well as their livestock globally, both in terms of severity and numbers. Intestinal parasitic infections are among the most common infections worldwide. It is estimated that a quarter of the world’s population is infected and that 450 million are ill as a result of these infections, the majority being children (GBD 2019 Diseases and Injuries Collaborators, 2020; Ahmed, 2023). Vector-borne diseases (viral, bacterial, or parasitic diseases that are transmitted by ectoparasites like ticks or mosquitoes) account for more than 17% of all human infectious diseases worldwide, causing more than 700,000 deaths annually (WHO, 2024a). One of the most known vector-borne parasitic diseases is malaria. The causative parasites are various *Plasmodium* species, which are transmitted by the ectoparasitic *Anopheles* mosquitoes. Malaria is responsible for an estimated 249 million cases globally and results in more than 600,000 deaths every year (WHO, 2023a). Other ectoparasites like *Aedes* mosquitoes transmit, among others, the pathogens which cause dengue fever.

The impact of parasites on pets is also severe, with 21% of dogs in the US found to be infected with intestinal parasites, particularly with the protozoan *Giardia*, pathogenic hookworms (*Ancylostoma caninum*), and whipworms (*Trichuris vulpis*) (Stafford et al., 2020). In Europe, every second domestic cat (50.7%) was found to be infected with at least one internal or one external parasite species (Beugnet et al., 2014). Ectoparasites like fleas, ticks, or mites were found in 29.6% of cats, with the mite *Otodectes cynotis* being the most frequently identified species (17.4%), followed by fleas (15.5%). Endoparasites like intestinal worms were identified in 35.1% of the cats, with the nematode *Toxocara cati* being the most commonly diagnosed species (19.7%). These parasites represent a lifelong risk for pets and can cause weight loss, exercise intolerance, general malaise, pain, discomfort, and in rare cases, illness that can be life-threatening if left untreated (Traversa, 2012).

Moreover, beyond the indirect benefit of disease-free pets on humans, there is a direct risk for humans for certain parasitic infections within their pets. More than 200 parasite species and the diseases they cause are of zoonotic characteristic, which means that they can also be transmitted to people and thus negatively affect the health of both people and pets. One example of a zoonotic disease that can be transmitted from dogs to humans is visceral leishmaniasis, also known as kala-azar. This disease is endemic in more than 65 countries, mostly distributed in Brazil, India, Ethiopia, Kenya, Somalia, South Sudan, and Sudan, but also on a subnational scale in endemic countries such as Italy, Greece, and Spain (Scarpini et al., 2022). The disease is caused by the zoonotic parasites *Leishmania donovani* and *L. infantum*, which are also found in dogs. *Leishmania* are transmitted by biting sandflies and cause up to 400,000 new cases annually worldwide with an estimated 50,000 deaths in 2010 (Lozano et al., 2012). Another example of a zoonotic disease is toxoplasmosis, which can be transmitted from cats to humans. Up to one-third of the human world population is infected with the protozoan parasite *Toxoplasma gondii* (Montoya and Liesenfeld, 2004).

Parasites also infect plants. The genus *Phytomonas* comprises phytopathogenic trypanosomatids that are transmitted by bugs (Hemiptera), which feed on plant phloem or fruit (Jaskowska et al., 2015). *Phytomonas serpens* parasitizes tomatoes, *P. staheli* infects coconut and oil palms, and *P. leptovasorum* infects coffee plants (Dollet, 1984). These parasites cause wilting and necrosis of the infected plant, which can lead to substantial economic losses in crops as important as those mentioned above. By far, the greatest economic impact of the phytopathogenic parasites is caused by soil-borne nematodes that attack plant roots. They include the lesion nematodes (*Pratylenchus* spp.), cyst nematodes (*Heterodera* spp.), and root-knot nematodes (*Meloidogyne* spp.). Together, these plant parasitic nematodes cause global crop yield losses estimated at $125 to $350 billion per year (Abd-Elgawad and Askary, 2015). The rice root-knot nematode *Meloidogyne graminicola* alone is responsible for an annual rice yield loss of 15% in Asia (Phan et al., 2020).

Here, we focus on parasites of humans, pets, and livestock. We provide an overview of the global impact of parasites and the benefits of parasite control, indicating the quantitative dimensions, the zoonotic risks, One Health aspects, and areas for improvement.

## 2 Impact of parasites on humans and public health

### 2.1 Impact of parasites on world history

The dramatic impact of parasites and the lack of control options on world history can be demonstrated in some cases retrospectively (Mäser, 2019). Subsequent to the introduction of the yellow fever virus and its competent vector, the ectoparasite *Aedes aegypti*, in the course of the inglorious transatlantic slave trade, this vector-borne disease became endemic in parts of America. When Napoleon invaded Saint-Domingue with 34,000 soldiers, it took less than a year that he had lost 24,000 men due to death and 7,000 severely ill, all due to the vector-borne yellow fever. Unaware of the nature of the disease and of any control measures, Napoleon was finally forced to give up the land “Louisiana” with the foreseen capitol Nouvelle-Orleans and to sell it all in 1803 for only $7/km^2^ to the United States of America. This largest land transaction ever increased the size of the US by approximately one-quarter and led to the renaming of today’s “New Orleans” (Marr and Cathey, 2013).

Napoleon was again defeated a few years later by an ectoparasite, the body louse. He went with an army of 400,000 men to conquer East Europe and had to turn back after half a year with only 40,000 men before the first battle had even started. The vector-borne disease typhus caused by *Rickettsia prowazekii* and transmitted by the body lice *Pediculus humanus corporis* had decimated his army to a large extent due to favorable conditions for the parasite as well as due to the cold climate, poor hygiene, and malnutrition (Peterson, 1995).

### 2.2 Morbidity and mortality of parasitic diseases in humans

The impact of diseases is often seen by the number of deaths associated with a particular pathogen. However, mortality alone does not give a complete picture of the burden of disease. According to the WHO, the overall burden is more accurately assessed using the “disability-adjusted life year” (DALY) measure. This time-based calculation combines years of life lost due to premature mortality and years of life lost due to time lived in conditions of less than full health, or years of healthy life lost due to disability (WHO, 2023b).

Until today, parasitic diseases have had a severe impact on human health regarding both causes of deaths and loss of years of healthy life. For example, in 2021, nearly half of the world’s population was at risk of malaria (WHO, 2024b), with 249 million cases, or 46 million DALYs in 2019 (GBD 2019 Diseases and Injuries Collaborators, 2020). Children under 5 years old account for approximately 80% of the more than 600,000 deaths every year.

Parasites are not only the causative agents of animal and human diseases but also often the main route for acquiring the infection. In general, vector-borne diseases, which include viral, bacterial, or parasitic diseases that are transmitted by ectoparasites like ticks or mosquitoes, account for more than 17% of all human infectious diseases worldwide, causing more than 700,000 deaths annually. One of the best-known vector-borne parasitic diseases is the aforementioned malaria. The causative protozoan parasites (various *Plasmodium* species) are transmitted by *Anopheline* mosquitoes. Other ectoparasites like *Aedes* mosquitoes transmit, among others, the pathogens that cause dengue fever and yellow fever. Transmitted by *Aedes aegypti*, yellow fever had an estimated incidence of over 100,000 severe cases in 2018 and a mortality of over 50,000 (Gaythorpe et al., 2021), in spite of the fact that a yellow fever vaccine has been available since 1937. More than 3.9 billion people in over 129 countries are at risk of contracting dengue, with an estimated 96 million symptomatic cases and an estimated 40,000 deaths every year (WHO, 2024c). Dengue and other emergent arboviruses are geographically spreading, largely due to the expansion of the mosquito vectors (Lwande et al., 2020). Future climate changes may even further extend mosquito distribution, such as the invasive *Aedes albopictus*, and thus increase the risk for vector-borne diseases (Laporta et al., 2023). Global warming also favors the expansion of vectors other than mosquitoes. Autochthonous populations of sandflies (*Phlebotomus mascittii*) have been reported from Vienna (Kniha et al., 2020) and Budapest (Bede-Fazekas and Trajer, 2015). In the Americas, triatomine bugs are spreading north and have established populations as far as Nebraska (Nielsen et al., 2021). Triatomine bugs are the vectors of *Trypanosoma cruzi*, the causative agent of Chagas disease. Due to its chronic and elusive nature, Chagas disease is a zoonosis that poses a particular threat to humans. Surveys of opossums and raccoons in Florida have shown infection rates with *T. cruzi* of approximately 50% as well as in peridomestic animals (Torhorst et al., 2023). Even more alarming, a citizen science project aimed at assessing the urban public health risk of Chagas disease in Caracas revealed *T. cruzi* infection rates of 80% in triatomines caught in domestic premises and a high proportion of human-positive bloodmeal analyses (Segovia et al., 2023).

Intestinal parasitic infections are among the most common infections worldwide. It is estimated that some 3.5 billion people are affected and that 450 million are ill as a result of these infections, with the majority being children. Of these, 1.5 billion people or 24% of the world’s population in 2021 was infected with soil-transmitted helminths, mainly roundworms (*Ascaris lumbricoides*), whipworms (*Trichuris trichiura*), and hookworms (*Ancylostoma duodenale*, *Necator americanus*) (Ahmed, 2023; WHO, 2023c). Intestinal parasitic infections are regarded as a serious public health problem, as they cause iron deficiency, anemia, growth retardation in children, and other physical and mental health problems.

In addition to the disease symptoms arising from the proliferation of parasites in our body, many parasitic infections are also associated with more indirect, chronic pathology such as autoimmune reactions in the case of Chagas disease (Soares et al., 2001), post-kala-azar dermatosis with visceral leishmaniasis (Zijlstra et al., 2003), or chronic inflammation in neurocysticercosis (Del Brutto, 2024). Thus, post-acute infection syndromes, well-known from long COVID, also occur in parasitosis.

Torgerson et al. (2015) have quantified the impact of 10 helminth infections plus toxoplasmosis on humans. The global burden of these 11 parasitic diseases accumulated to 8.78 million DALYs, of which 6.64 million DALYs were estimated to be via contaminated food (Torgerson et al., 2015). Foodborne toxoplasmosis was found to have a rather high burden on humans in terms of DALYs (825,000), mainly due to years lived with disability.

## 3 Impact of parasites on pets

### 3.1 Impact on pets, their owners, and global economics

One of the most important endoparasites affecting the health of companion animals, health, from both a pathologic and an economic perspective, is *Dirofilaria immitis*, the filarial parasite that causes heartworm disease. Infections in dogs and also cats with heartworms result in severe diseases that can lead to substantial clinical signs and can be fatal (Bowman and Wu, 2022). Initially, such clinical signs may include a persistent cough, reluctance to exercise, fatigue after moderate activity, decreased appetite, and weight loss. With the progression of the disease, dogs may develop heart failure and those with large numbers of heartworms can develop a sudden blockage of blood flow within the heart, leading to a life-threatening form of cardiovascular collapse. At this stage of the disease, prompt surgical removal of the heartworm blockage is the only way that dogs can survive (Bowman and Drake, 2017).

Treatment of an established *D. immitis* infection in dogs requires a prolonged regimen of chemotherapy, exercise restriction, and sometimes even surgery. Therefore, the current practice is to control heartworm disease through prevention, with drugs from the chemical class of the macrocyclic lactones. However, successful prevention of heartworm disease in dogs may be compromised by drug-resistant *D. immitis* (Bourguinat et al., 2015; Bowman and Wu, 2022). In endemic areas, it is recommended to pursue prevention on a year-round basis. The American Heartworm Society^1^ recommends that pets should be tested every 12 months for heartworm and that pets should receive heartworm preventives 12 months a year.

Heartworms are transmitted as larval stages by various mosquito species that acquire the *D. immitis* microfilarial stage in a blood meal from an infected host (dogs, cats, and others). *Dirofilaria immitis* infections in dogs and cats have been identified throughout the world in tropical and temperate regions (Simon et al., 2009; Genchi and Kramer, 2020; Noack et al., 2022).

On top of the emotional impacts for pet owners due to the concern for their pets being infected and during treatment, there is a substantial economic impact of heartworm disease on the pet owner. The cost can be calculated by separating cost components into the cost of prevention (heartworm medication), the cost of treatment, and the opportunity cost of treatment. From a geographic standpoint, estimates can be separated on the impact within the key heartworm countries (USA, Australia, Japan, Italy, Spain, and Canada) and the rest of the world in aggregate. Publicly available calculations provide an estimated total global cost of heartworm disease of US$2.47 billion (Klug and Drake, 2022). The cost of prevention makes up 93% or US$2.30 billion of this total, with the cost of treatment representing 6% or US$146 million and the opportunity cost to the pet owner of 1% or US$24.6 million. From a geographic standpoint, the USA accounts for 65% of the global costs, while one in three dogs is under heartworm prevention with an average of 8.6 doses per year (Drake and Wiseman, 2018). Year-round prevention is essential for controlling the disease in individual dogs and to avoid outbreaks in endemic areas, since one bite of an infective mosquito is sufficient to get the infection going.

There are more than 470 million dogs and 370 million cats worldwide^2^. The number of dogs at risk for heartworm infection is approx. 148 million in Asia, approx. 98 million dogs each in Latin America and in Europe, approx. 80 million in North America, and approx. 50 million in Africa. In North America, approximately 1% of the tested dogs are positive, resulting in approximately 100,000 dogs being diagnosed with heartworm infection annually (Companion Animal Parasite Council https://www.capcvet.org/, accessed in October 2023). More than 200 million dogs live in North America, Europe, Australia, and Japan, where prophylaxis and treatment probability are expected to be high (Noack et al., 2022). Thus, *D. immitis* constitutes one of the most attractive markets for anti-infective drug development (Klug and Drake, 2022). This contrasts with the closely related human pathogenic filariae such as *Onchocerca volvulus*, *Wucheria bancrofti*, and *Brugia malayi*, causative agents of river blindness and lymphatic filariasis, respectively, which are important but neglected tropical parasites. These diseases primarily affect low-income populations and are a continuous barrier to socioeconomic development (Boatin et al., 2022). There is an opportunity here for One Health drug repurposing (Hamid et al., 2024), as exemplified by the Mectizan Donation Program (Colatrella, 2008).

### 3.2 One Health aspects—zoonotic risk

The term “One Health” first appeared in the biomedical literature in 2005 concerning the potential for strengthening health systems (Zinsstag et al., 2005; Cassidy, 2017). Subsequently, the correlation and deep connections between human and animal health and the need for an interdisciplinary and collaborative approach to respond to emerging diseases were clearly outlined and proposed^3^ (Mackenzie et al., 2014), although the principles of the One Health concept originated several decades ago as “One Medicine, One World” (Cassidy, 2017). The concept has not been applied to the study of parasites as frequently or intensively as might be desired (Zinsstag et al., 2020). Obvious indicators for a link between human and animal diseases are a) the origin of the pathogen, b) shared geographic or microhabitats, and c) a zoonotic character of the disease. Noteworthy, at least 60% of human diseases are multi-host zoonoses (Cleaveland et al., 2001).

Pathogens that can be transmitted from animals to humans are causing over 200 known types of zoonotic diseases. These pathogens include one single protein (Bartz et al., 2024), various bacteria, viruses, fungi, and a large number of parasites (Weiss, 2008). In fact, the majority of the classic parasitic diseases due to protozoa (various unicellular parasite species causing leishmaniasis, giardiasis, toxoplasmosis, trypanosomiasis, amoebiasis, giardiasis, a.o.), cestodes (various tapeworms causing echinococcosis, taeniasis, a.o.), trematodes (various flukes causing schistosomiasis, clonorchiasis, fascioliasis, paragonimiasis, a.o.), nematodes (various roundworms causing filariasis, trichostrongyliasis, strongyloidiasis, a.o.), pentastomids, or arthropods are zoonotic. Some of these diseases such as cryptosporidiosis, toxoplasmosis, and leishmaniasis have gained importance as human pathogens because they can cause severe diseases, particularly in patients with immune suppression (Weiss, 2008). Also, parasites that cannot further develop in humans can cause pathology, e.g., the larvae of the dog nematodes *Ancylostoma caninum* (cutaneous larva migrans) and *Toxocara canis* (visceral or ocular larva migran) (Marques et al., 2012; Sharma et al., 2015).

Many zoonoses are not restricted to the tropics and impose a burden also in the global North. The fox tapeworm *Echinococcus multilocularis*, for instance, is present throughout the Northern Hemisphere (Deplazes et al., 2017). Alveolar echinococcosis is a life-threatening infection in humans. More harmless, but unpleasant nevertheless, is an infection with the fish tapeworm *Diphyllobothrium latum*, which a wedding party in Switzerland had to experience first-hand after the consumption of marinated raw perch from Lake Geneva (Jackson et al., 2007).

Zoonotic diseases represent an increasing public health problem around the world due to our close relationship with animals including companion animals like dogs and cats. There are increasing numbers of cases of zoonotic infections on the background of overpopulation, disruptions due to military action, mass migrations of populations, and even worldwide tourism (Weiss, 2008). The five major parasitic zoonoses when calculating the human burden of diseases following the WHO terms of DALYs are cryptosporidiosis (8.37 million DALYs), intestinal nematode infections (5.16 million), leishmaniasis (3.32 million), schistosomiasis (3.31 million), and lymphatic filariasis (2.78 million) (Pisarski, 2019).

Example 1 for a zoonotic disease in dogs: One of the major parasitic zoonoses with more than 3 million DALYs is leishmaniasis (Hotez et al., 2014), a spectrum of diseases with various clinical symptoms and caused by approximately 20 different *Leishmania* species (Cecilio et al., 2022). There are three main forms of leishmaniases. Visceral leishmaniasis is the most serious form because it is usually fatal without treatment. Other diseases are the most common cutaneous leishmaniasis and the mucocutaneous leishmaniasis. All pathogenic leishmania parasites are transmitted by approximately 100 different ectoparasitic sandfly species during the bite and blood feeding of the infected females. Since sandflies choose their hosts mostly by availability, it is not surprising that some *Leishmania* species like *Leishmania infantum* or *L. braziliensis* are found in humans and their nearby living dogs, increasing the risk of the zoonosis to be spread (Cecilio et al., 2022). Apparently, the prevalence is increasing, and the World Health Organization estimates 700,000 to 1 million new cases to occur annually^4^.

The zoonotic potential of leishmaniasis is a threat to humans even in the absence of dogs as reservoir hosts. An outbreak of leishmaniasis in Fuenlabrada in the metropolitan area of Madrid in 2010 was traced back to hares. The construction of a protected, irrigated park had boosted the proliferation of sandflies as well as hares, 30% of which were infected with *L. infantum* (Aguado et al., 2013). This created a peri-urban transmission cycle that spilled over to human visitors when they got bitten by the sandflies.

Example 2 for a zoonotic disease in cats: The protozoan parasite *T. gondii* infects virtually all warm-blooded animals, including humans. Up to one-third of the global human population is infected with *T. gondii* (Montoya and Liesenfeld, 2004). Moreover, the *T. gondii* disease burden has been ranked among the highest of parasitic diseases (Djurkovic-Djakovic et al., 2019) with an estimated equivalent of 1.2 million DALY for >190,000 annual cases of congenital toxoplasmosis (Torgerson and Mastroiacovo, 2013), including severe cases of congenital infection such as hydrocephalus, microcephalus, or hydrops fetalis (Konstantinovic et al., 2019). In addition, foodborne toxoplasmosis with 10.3 million cases per year adds 825,000 DALYs to the disease burden (Torgerson et al., 2015). People typically become infected by three principal routes of transmission: animal-to-human (zoonotic), foodborne, or mother-to-child (congenital). Cats (or other animal species of the Felidae family) are the final host of the *T. gondii*, and after the sexual phase of the parasites in the cat, these are shedding the infective oocyst stage in the feces. People can then be infected by accidental ingestion of oocysts or by ingestion of tissue forms (cysts consisting of bradyzoites) in contaminated food. Although generally mild and self-limiting in immunocompetent individuals, *T. gondii* infection may cause life-threatening disease in the fetus and in the immunosuppressed host (Djurkovic-Djakovic et al., 2019). Furthermore, studies on elderly male people indicate that a performance reduction of approximately 35% in working memory is caused by an otherwise asymptomatic infection with *T. gondii* (Gajewski et al., 2014). The impairment of memory functions in *T. gondii*-positive seniors was accompanied by a self-reported decreased quality of life. To reduce the disease burden of toxoplasmosis in humans, interventions are needed in animal reservoirs. However, Bonacic Marinovic et al. (2019) showed with a disease dynamics compartmental model for a hypothetical cat vaccine that only a few cats were allowed to stay unvaccinated to substantially reduce *T. gondii* oocysts in the environment. Thus, reducing oocyst-acquired human infections by vaccination coverage of cat populations seems unrealistic (Bonacic Marinovic et al., 2019) and defining effective intervention strategies to control zoonoses at the animal–human interface is rather complex (Mummah et al., 2020). There is a vaccine for sheep; however, there is still no vaccine to prevent toxoplasmosis in humans, while the rather small repertoire of effective drugs (pyrimethamine, sulfadiazine, sulfadoxine, clindamycin, spiramycin) is additionally limited by important side effects (Djurkovic-Djakovic et al., 2019). Today, screening pregnant women appears to be the feasible way to monitor and treat congenital toxoplasmosis. A recent calculation in France showed that screening every pregnant woman for toxoplasmosis would have saved €148 million in addition to reducing or eliminating the devastating physical and emotional suffering caused by *T. gondii* (Sawers et al., 2022).

Example 3 for a zoonotic disease in ruminants: Zoonotic parasites exist not only in pet animals but also in livestock. The unicellular parasite *Cryptosporidium parvum* is not only causing the most important disease of young ruminant livestock but is also responsible for the second biggest cause of infant diarrhea and death in Africa and Asia (Liu et al., 2012; Striepen, 2013). Cryptosporidiosis is endemic in cattle worldwide and is responsible for more than 50% of neonatal enteritis in calves (Thomson et al., 2017). The economic losses associated with bovine cryptosporidiosis include the cost of treatment and management of enteritis, reduced feed conversion and production efficiency, and losses due to animal death, determined to be at least 34 British pounds per calf. The zoonotic implication was demonstrated in 2010 by Liu et al. (2012), who showed that diarrhea accounted for 10.5% of the 7.6 million deaths of children under the age of 5, with *C. parvum* and *C. hominis* as the major causative agents.

### 3.3 One Health aspects – psychological and physical benefits for the pet owners

The direct health benefits of vector control for animals are often obvious and profound. However, the indirect human health benefits of control or prevention of parasitic diseases in pets should be included as well when assessing the impact and benefits of controlling parasites. In many parts of the world, companion animals, particularly dogs and cats, have been integrated into family life, sometimes to the extent that pets are considered to be family members. The pet–owner bond, particularly as it relates to the wellbeing of these animals, contributes to a large extent to the overall life experience of the people involved, and as such, healthy pets can contribute to human health by providing many positive psychological and physical benefits for their owners (McConnell et al., 2011; Cordaro, 2013; Mubanga et al., 2017). The owners of healthy pets do not need to worry about pet ill-health but are free to enjoy and live with their pets including common physical exercises. Significant health benefits are apparent in companion animal owners who are free from worry over possible parasitic infections of their animals and pet ill-health.

## 4 Impact of parasites in livestock

### 4.1 Endoparasites in ruminants and poultry

Until today, livestock animals like cattle, small ruminants, swine, and chicken are a major source of proteins for humans. There are numerous studies on the prevalence of parasites, mainly gastrointestinal nematodes, for example in sheep. As an average from publicly available reports, approximately three of four sheep are infected with one or, more likely, multiple species of these nematodes. Asmare et al. (2016) determined a prevalence rate of 76% in 9,407 sheep and 3,478 goats in Africa, Tachack et al. (2022) found a prevalence rate of 88% in South America, and Idris et al. (2012) found a rate of 63% in 3,924 lambs in Germany (Idris et al., 2012; Asmare et al., 2016; Tachack et al., 2022). Grzonka et al. (2000) reported even a 100% infection rate in sheep of 40 flocks in Germany (Grzonka et al., 2000). In addition to gastrointestinal nematodes, the majority of sheep is also parasitized by other parasites. In one study conducted in Germany, only 11.4% of the lambs were free of *Eimeria* oocysts (Idris et al., 2012).

The negative impact of gastrointestinal nematodes on sheep was quantified by Mavrot et al. (2015) in a meta-analysis of 88 studies. When comparing parasite-free and infected animals, the results indicated that in parasite-infected animals, the production in terms of weight gain, wool, and milk is reduced to 77%, 90%, and 78%, respectively, in comparison to parasite-free animals (Mavrot et al., 2015).

The control of parasitic worm infections was shown to lead to impressive social benefits for farmers. In the McKinnon project with four farms in Australia, worm control in combination with scientific-based recommendations for general farming processes over a period of 7 years resulted in a substantial improvement in the financial and physical performance of the farmers (Lean et al., 1997). The mean gross farm income of the four farms steadily rose from 86% of the average benchmark farm before the adoption of recommendations to an average of 155%. During the same period, net farm income rose from 70% to 207% of the average benchmark farm.

However, gastrointestinal nematodes are not the only parasitic infection in livestock. Coccidiosis in cattle and poultry is caused by various apicomplexan parasites like *Eimeria*, *Isospora*, *Neospora*, *Cryptosporidium*, and others. Many species are responsible for reduced growth, mortality, and reduced meat quality in chicken and cattle. The current costs of control for the cattle farmers, considering the number of calves borne, mortality rates, estimated profits, feed inefficiency, and other parameters, have been estimated to sum up to €490 million for the US and Europe for cattle (Klug, 2017). The global poultry industry appears to be even more affected by coccidiosis. Annual losses have been estimated to exceed US$3 billion (Noack et al., 2019). At present, poultry production is optimized by using anticoccidials, benefiting all three pillars of sustainability, namely, social (bird health, animal welfare, and food safety), economic (production efficiency), and environmental aspects (Kadykalo et al., 2018).

### 4.2 Impact of the ectoparasitic “sea lice” on protein production in aquaculture

Fish is an excellent protein source with the additional value of polyunsaturated fatty acids (omega-3 fatty acids). Capture fishery is unable to satisfy the growing demand for fish; thus, farmed fish became a valuable alternative. Aquaculture has been the fastest-growing food production sector since 1970 (Guo and Woo, 2009), but with associated problems like parasitic pathogens. The fish-parasitic “sea lice” are not lice but actually belong biologically to the crustacean organisms (Guo and Woo, 2009). Two main parasitic species, *Lepeophteirus salmonis* and *Caligus rogercresseyi*, are threatening fish farming, particularly salmon farming in the Northern (Norway, Scotland, Ireland, Faeroes, Canada, United States) and Southern Hemisphere (mainly Chile). These ectoparasites have a rather complicated life cycle, with 8 or 10 different life stages. While the free-swimming larvae are subject to currents, it is the copepedid and the chalimus stages which need to locate, contact, and attach to a suitable host like salmon (Brooker et al., 2018). The commonly called “sea lice” suck blood and damage fish from the outside in. However, the damage is not just on the fish but also on the public perception of fish farming. Declines in wild salmonid populations have been linked between sea lice in salmon farms and this decline (Costello, 2009).

Treatment relies on few parasiticides only (Brooker et al., 2018), and thus, it was inevitable that with higher demand and increasing biomass of fish and amount of parasiticides applied (Bravo and Treasurer, 2023), severe drug resistance developed toward the main drugs (azamethiphos, the pyrethroids, and the macrocyclic lactone emamectin) and spread rapidly in 2008–2009 (Hannisdala et al., 2020). Costs for treatment were calculated for Norway, the largest salmon-producing country, and amounted to 3.4 billion NOK per year (approx. €290 million) in 2014 for 1.272 tons produced salmon and 5 billion NOK (approx. €426 million) in 2015 for 1.303 tons produced salmon (Brooker et al., 2018). Estimates for the global costs to control range from €305 million (Costello, 2009) to €768 million in 2015 (Brooker et al., 2018).

*Spironucleus salmonicida* is a unicellular parasite that is related to the mammalian intestinal parasite *Giardia intestinalis*. However, *S. salmonicida* can escape from the gastrointestinal tract of infected fish and cause a systemic, lethal infection. As with the sea lice, *S. salmonicida* dwells in fish farms due to the large number of hosts and their often poor immune status, and it has caused substantial losses to the Norwegian salmon industry.

## 5 Concluding remarks

All of the above examples show just a minor picture of how parasites impact the health of pets and livestock, human health, risk of infection, and food and environmental safety and security. Growing zoonotic and chronic animal diseases have a significant impact on humans. Thus, control of parasites is an essential need for animal and human health (Kaminsky et al., 2013). The market for parasiticides was quantified to be €7 billion in sales in 2018, covering 23% of the market share of the overall animal health market (Selzer and Epe, 2021) and is estimated to grow approximately 7% per year as per forecast 2023–2032^5^. The primary driver of this market is the increase in the cases of zoonotic as well as chronic illness cases. The key needs for improved control are as follows: a) convenience of treatment for pets and livestock (easy to apply, long duration a.o.), b) effectiveness against drug-resistant parasites (Nixon et al., 2020) and reduced costs for livestock, and c) control measurements that are environmentally friendly.

## Footnotes

1
https://www.heartwormsociety.org/.

2
https://www.statista.com/statistics/1044386/dog-and-cat-petpopulation-worldwide.

3
https://www.oneworldonehealth.org/.

4
https://www.who.int/news-room/fact-sheets.

5
https://www.precedenceresearch.com/veterinarymedicine-market.
